# Endothelial Heterogeneity in Development and Wound Healing

**DOI:** 10.3390/cells10092338

**Published:** 2021-09-07

**Authors:** David B. Gurevich, Deena T. David, Ananthalakshmy Sundararaman, Jatin Patel

**Affiliations:** 1 Department of Biology & Biochemistry, Faculty of Science, University of Bath, Claverton Down, Bath BA2 7AY, UK; dbg29@bath.ac.uk; 2 Cardiovascular Diseases and Diabetes Biology, Rajiv Gandhi Centre for Biotechnology, Thycaud, Thiruvananthapuram 695014, India; tdaviddeen@gmail.com (D.T.D.); ananthalakshmys@rgcb.res.in (A.S.); 3 Cancer and Ageing Research Program, School of Biomedical Sciences, Queensland University of Technology, Brisbane 4102, Australia

**Keywords:** heterogeneity, endothelium, neovascularisation, angiogenesis, EndMT

## Abstract

The vasculature is comprised of endothelial cells that are heterogeneous in nature. From tissue resident progenitors to mature differentiated endothelial cells, the diversity of these populations allows for the formation, maintenance, and regeneration of the vascular system in development and disease, particularly during situations of wound healing. Additionally, the de-differentiation and plasticity of different endothelial cells, especially their capacity to undergo endothelial to mesenchymal transition, has also garnered significant interest due to its implication in disease progression, with emphasis on scarring and fibrosis. In this review, we will pinpoint the seminal discoveries defining the phenotype and mechanisms of endothelial heterogeneity in development and disease, with a specific focus only on wound healing.

## 1. Introduction

Establishment and maintenance of a functioning vascular network is essential for initial embryonic development, as well as subsequent postnatal life, providing vital nutrients to tissues [1]. The fundamental building blocks of this system are endothelial cells, which comprise the inner lining of the vasculature and form a tightly regulated barrier across which oxygen, metabolites, circulating cells and soluble factors are trafficked [2]. As such, endothelial cells are crucial to the control of key vascular functions, including vessel permeability that regulates bloodstream–tissue exchanges, vasomotor tone and blood fluidity [3]. Moreover, endothelial cells interface with other lineages important in vessel formation and maintenance, for instance pericytes, smooth muscle cells and immune cells [4]. However, the single cell layer endothelium is anything but identical, displaying a remarkable heterogeneity and plasticity that is only now becoming elucidated [5,6]. A combination of genetic sequencing technologies, lineage tracing and live imaging experiments in mouse, zebrafish and tissue culture have begun to inform us how this heterogeneity drives endothelial cell phenotypes and behaviours to result in proper vascular development and maintenance [7,8,9]. The development in understanding endothelial heterogeneity has also had profound impacts in understanding the biology of vasculogenesis/angiogenesis in pathologies such as cancer, where vasculature if crucial for tumour progression (reviewed by De Palma et al.) [10]. More recently, newly developed technologies such as genomic profiling techniques via single cell RNA sequencing hold enormous promise to revolutionise our understanding of the transcriptional and proteomic changes occurring at the levels of single cells and how these impact on vessel formation and function [8,11]. Additionally, our understanding of endothelial plasticity has grown significantly, especially with the notion of endothelial to mesenchymal transition (EndMT). EndMT is a crucial driver in the formation of important anatomical structures during foetal development [12]. However, in the adult system it has been implicated in disease progression due to vascular damage and scar tissue deposition, in particular during wound healing [13,14]. In this review, we will focus on endothelial cell heterogeneity and plasticity between lineages, developmental stages, and in health and disease, with a specific focus on wound healing.

## 2. Developmental Heterogeneity of Endothelial Cells

**Figure 1:**
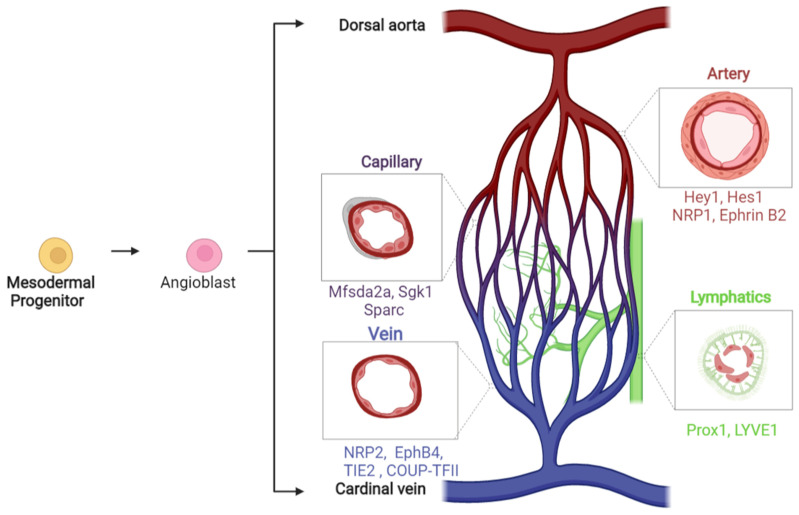
Schematic illustrating the major markers of endothelial diversity generated throughout vascular development, as mesodermal progenitors give rise to angioblasts that ultimately differentiate into endothelial cells with specific arterial, venous, lymphatic and capillary identities. Understanding of these developmental markers and mechanisms has built up from decades of fundamental research using avian, rodent, fish and human tissue culture models [8,17,18,19,20,21,22,23,24]. Image produced in BioRender.

Arising predominantly from the lateral plate mesoderm (LPM), endothelial cells are the first component of the developing vasculature [15,16]. Endothelial cell heterogeneity and a myriad of transcription factors is evident even at the earliest stages of vascular development known as vasculogenesis, where the major vessels are formed from angioblasts, with the resultant endothelial cells already assuming arterial, venous or lymphatic identity (Figure 1) [17,18,19,20,21,22,23,24]. Live imaging and lineage tracing experiments in the zebrafish revealed that the angioblasts involved in vasculogenesis already possess differing potentials, with the medial angioblast pool migrating first and primarily generating endothelial cells that form the dorsal aorta (DA), while the lateral angioblasts migrate later and generate endothelial cells forming the posterior cardinal vein (PCV) [25,26]. Mammalian arteriovenous differentiation follows a similar sequence, with mouse studies demonstrating that the dorsal aortae and cardiac precursors are the first intra-embryonic vascular components to be formed, shortly followed by vein primordia [27]. These distinct populations are regulated by specific signalling pathways, with arterial cells undergoing angiogenesis in response to Vascular Endothelial Growth Factor (VEGF) signalling, but not bone morphogenetic protein (BMP) signalling, while venous endothelial cell sprouting is conversely regulated by BMP but is VEGF-independent [28,29]. DA-fated angioblasts were also shown to differ from PCV-fated angioblasts by having increased Erk signalling and early Notch activity upon departure from the LPM [30,31]. However, DA-derived angioblasts with high Notch activity can contribute to endothelial cells forming both arterial and venous vessels at slightly later developmental stages, such as the intersegmental vessels (ISVs) during primary angiogenesis in the zebrafish [31]. Moreover, studies in mouse and zebrafish show a particular conservation of endothelial cell plasticity at this early developmental stage, whereby endothelial cells can move between arterial and venous vessels following initial medial migration [32,33]. Beyond this, lineage tracing experiments in the zebrafish reveal that lymphatic cranial vessels—unlike all other lymphatic vessels that arise from venous sources—are derived from ventral aorta arterial angioblasts in later migration waves, and non-venous origins for various lymphatic vessels have also been implicated in mouse studies [34,35,36].

The establishment of individual vessels highlights that endothelial cell heterogeneity extends beyond different organs. For example, the structure of the endothelium for various types of networks varies enormously: arteries and veins comprise an uninterrupted, continuous layer of cells, while capillaries have a variety of tissue-dependent organisational options, including discontinuous and fenestrated arrangements. This structural heterogeneity is mirrored in gene expression, with experiments performed on different mammalian arteries showing distinct genetic profiles to each other [37]. Endothelial cell heterogeneity can be seen in different vessels within the same tissue and even adjacent endothelial cells within the same vessel [38]. Studies have revealed the importance of interactions between Notch and VEGF signalling pathways in defining tip cell and stalk cell identities. Endothelial cells are initially induced to sprout from the DA via VEGF signalling, stimulating Notch signalling in these newly determined tip cells, thus in turn suppressing VEGF signalling in the trailing stalk cells [39,40,41,42]. Although VEGF signalling is indeed implicated as the most crucial signalling input for endothelial cells, additional factors are also important in regulating key endothelial cell functions and behaviours. For example, genetic expression studies revealed how certain genes—such as transmembrane 4 L six family membrane 18 (tm4sf18)—are restricted to these actively sprouting endothelial cells, and function as amplifiers of VEGF signalling for specific subsets of endothelial cells, to fine-tune vessel formation [43]. Calcium (Ca^2+^) oscillations have also been shown to correlate with specific endothelial cell behaviours, with maintenance of Ca^2+^ activity restricted only in sprouting tip cells in both zebrafish and in mammalian tissue culture [44,45]. Further examples of heterogeneity mechanisms are seen in live imaging of endothelial cell divisions, showing that tip cells undergo a cell division event during sprouting that results in asymmetric cytoplasmic distribution and subsequently a rapid re-acquisition of tip/stalk identities [46]. Asymmetric division is also a central to the generation of lymphatic vessels: by following the expression of *Prox1*, a key marker of lymphatic endothelial cells in vertebrates, live imaging studies in zebrafish have revealed a division event by Prox1 positive progenitors in the PCV that gives rise to one daughter cell that upregulates Prox1a and migrates dorsally to form lymphatic sprouts, and one daughter cell that downregulates prox1a and stays in the PCV [18,47,48]. Finally, endothelial cell plasticity and heterogeneity accommodates, responds to, and integrates with broader developmental changes. As vascular networks expand, variations to flow patterns and shear stress results in morphological changes to the individual endothelial cells that comprise the DA in zebrafish, driving alterations to the overall vessel architecture and narrowing of the DA diameter over time [49]. Studies of mammalian endothelial cells have revealed that flow is a key component of how endothelial cells align along different regions of a vessel, whether in an axial or non-axial pattern, and that this organisation can be remodelled in response to flow alterations [50]. The approaches that angiogenic sprouts use to anastomose and form a functional lumen also differs between vascular beds and individual vessels. Live imaging studies demonstrating that endothelial cells can engage in a variety of mechanisms from fusion of intracellular vacuoles to collective migration and ensheathment of areas of blood flow, to a process driven by dynamic membrane protrusions termed “inverse blebbing” [26,51,52]. Importantly, this diversity of lumenisation methods provides the capacity to establish a heterogeneous vessel architecture that can include unicellular and multicellular tubes in a variety of arrangements, encompassing all possible permutations required by mature tissues [53].

## 3. Endothelial Cell Heterogeneity in Homeostasis and Repair

In homeostatic adult tissue, endothelial cells represent some of the longest-lived cells in the organism, and their continued barrier and regulatory functions within the vessel wall are essential for proper functioning of the vasculature. To facilitate this, endothelial cells maintain a remarkable plasticity, vital for adapting their behaviour and identity to the distinct and changing requirements of the tissue they supply, and this is reflected in structural and molecular heterogeneity observed in particular vascular beds [54]. Evidence for endothelial cell heterogeneity being a fundamental property of endothelial cells in developed organisms is provided from studies on the hagfish, which represents the oldest extant vertebrate that still has an endothelial cell-lined closed circulation, and which displays endothelium that is molecularly, structurally and functionally heterogeneous [55]. Endothelial cells must respond to a variety of different types of changes in the tissue microenvironment, from changes to metabolism, mechanical forces and interactions between endothelial cells and matrix components as well as other cells, and organotypic growth factors (reviewed in [54]). Specific examples of this heterogeneity in mammalian contexts include fetoplacental endothelial cells that function in chronically low oxygen environments, endothelial cells present in the kidney that must contend with both hypoxia and hyperosmolarity, and liver endothelial cells that engage in clearance of harmful substances as well as modulation of immunoregulatory mechanisms [56,57,58]. Perhaps to accommodate this, endothelial cell organ and tissue specific heterogeneity is observed at early stages of human development, with endothelial cells profiled at three months gestation showing distinct tissue specific gene expressions for kidneys, liver, heart and lungs [59]. An additional study by Jambusaria et al. highlighted that this tissue-specific endothelial cell heterogeneity is maintained in adult homeostasis, and becomes even more integrated into the expression profile of endothelial cells [60]. Using single cell RNAseq to extensively compare endothelial cells from various mouse vascular beds, they demonstrated a reciprocity in specific endothelial cells expression profiles, revealing an intriguing property of endothelial cells to express ‘parenchymal’ genes—such as contractile genes in cardiac endothelium, or synaptic vesicle genes in brain endothelium [60].

Additionally, endothelial progenitor cells, whether vascular resident or found in circulation from the bone marrow, play an essential role in differentiating down a defined endothelial hierarchy to give rise to mature endothelial cells, allowing for vasculogenesis at organ specific sites [61,62]. Nolan et al. also elegantly demonstrated this following transplantation of endothelial cells derived from embryonic stem cells. Here, transplanted endothelial cells engrafted into regenerating tissues and acquired genetic features specific to endothelial cells of that organ bed [5]. A recent study by Palikuqi et al. showed the development of an organ-on-a-chip VascularNet model that allows the study of crosstalk between endothelial cells and parenchymal cells, assisting in identifying endothelial heterogeneity [63]. In vitro assessment of endothelial heterogeneity has also been extensively studied, using tools such as embryonic stem cells, or induced pluripotent stem cells to differentiate into endothelial cells and/or the isolation of primary endothelial progenitors isolated from human tissue. These studies have identified key pathways, transcription factors and molecular mechanisms that drive arterial, venous and lymphatic specification [64,65]. Importantly, much of this heterogeneity is dependent on the interactions between endothelial cells and their microenvironment: while microarray studies on various cultured human endothelial cell types showed maintenance of specific expression profiles following multiple passages, other gene expression studies revealed that up to half of the specific vascular bed signatures were lost after in vitro culture [66,67]. Herein lies the complexity of in vitro assessment of endothelial heterogeneity but also the failure to potentially reproduce results in different lab environments. Understanding this biology in an in vitro setting provides excellent insight into potential in vivo endothelial differentiation and potential cell–cell interactions in co-culture conditions; however, this needs confirmation using powerful genetic modelling in mice and zebrafish.

In healthy tissue, most transfer of material between blood and tissue occurs within the capillaries, where the density of the membrane-bound vesicles utilised for this transport known as caveolae is greatly enriched compared to arteries or veins (reviewed in [68]). However, in the context of inflammation, permeability to fluids, solutes and leukocytes is induced due to changes in the barrier function of these normally impermeable vessels, mediated by alterations to endothelial cell behaviour and function. This includes the regulation of pro- and anti-coagulant production, expression of E- and P-selectins to induce leukocyte rolling and expression of vascular cell adhesion molecules such as VCAM and ICAM to induce leukocyte adhesion and recruitment (reviewed in [6]). Intriguingly, while inflammation seems to activate a broadly similar inflammatory response, there is a persistent tissue-specific heterogeneity of endothelial cells in response to inflammation [69]. Examples include particular upregulation of P-selectin—a key mediator of platelet activation and aggregation—in heart and brain, which are tissues that are susceptible to thrombotic events, whereas lung endothelial cells have a marked upregulation of specific chemokines such as Cxcl1 and Cxcl9 [60]. Single cell transcriptomic experiments have also uncovered endothelial cell subsets that express genes involved in immune surveillance and interferon signalling across multiple tissue types, although the exact pro- or anti-inflammatory function of these subtypes and key identifying markers remain to be determined [8,70].

Endothelial cell heterogeneity and plasticity is further observed in instances of tissue damage. Studies of wounds in the zebrafish trunk show that blood vessels in the regenerate are chimeric, with contributing endothelial cells expressing markers of arterial or venous lineages [71]. Live imaging of zebrafish fin regeneration revealed a specific capacity for venous-derived cells to migrate into and integrate with remodelling arteries [72]. The chemokine receptor Cxcr4a is indispensable for this venous-to-arterial transformation to occur in the zebrafish, and the importance of CXCR4 appears conserved in mammals where CXCR4 expression is observed in the tip cells of developing mouse retinal arterial vessels [72,73]. In a recapitulation of development, both VEGF and BMP signalling have been shown to be highly upregulated in immune cells, fibroblasts and keratinocytes within mouse wounds, and these pathways appear to have a distinct role in modulating wound angiogenesis by differentially regulating endothelial cell tip vs. stalk cell identity in culture [74,75,76,77,78,79,80]. Additionally, live imaging of skin wound healing studies identified the migration of tissue resident endovascular progenitors (EVP) into the centre of wound granulation tissue and subsequent differentiation. EVPs have been identified to reside in vasculature and can give rise to mature endothelial cells, forming a vascular network independent of angiogenesis in ischemic situations of skin would healing [7,81]. Increased angiogenesis and neovascularisation has also been observed in skin wound healing using human EPC as a therapy, selectively blocking αVβ3 integrins, endothelial extracellular vesicles, antioxidant administration such as bee venom and also the activation of the PI3-kinase/Akt pathway [82,83,84,85,86,87,88]. As a further demonstration of endothelial cell plasticity, mouse studies of myocardial infarction have shown that endothelial cells can engage in localised clonal expansion in response to injury, allowing for a rapid expansion of the existing vessel architecture [89]. More extreme examples of endothelial cell heterogeneity were seen in transcriptomic and epigenomic characterisations of endothelial cells from mouse carotid arteries following partial ligation, which demonstrated a reprogramming towards a phenotype expressing macrophage markers, and indeed disturbed flow also induced this immune cell-like phenotype in cultured human aortic endothelial cells even in the absence of macrophages [90].

## 4. Endothelial to Mesenchymal Transition (EndMT)

Endothelial cells can undergo transdifferentiation, highlighting their remarkable plasticity. During embryonic development, diverse cell types like the hematopoietic stem cells, cardiac valve progenitors and even cardiac fibroblasts and smooth muscle cells arise from specialised endothelial cells [91]. Endothelial cells also undergo endothelial to mesenchymal transition (EndMT) in pathological contexts, for example in response to unique signals from their niche to transdifferentiate into fibroblasts contributing to cardiac fibrosis [92,93]. Indeed, the EndMT transition is central to wound healing as the pro-inflammatory and hypoxic wound site triggers EndMT in migrating endothelial cells from the local vascular bed [81,94]. Aberrant endothelial transdifferentiation, both delayed or advanced differentiation, can give rise to unstable vessels at the wound site resulting in excess scar tissue formation [81].

EndMT is characterised by the loss of the regular cobblestone morphology of the endothelium and the acquisition of mesenchymal spindle shape. Numerous studies have identified that when endothelial cells begin to transition, several intermediate phenotypes are formed and proceeds with the loss of endothelial markers like CD31 (PECAM), ZO1 and VE Cadherin, which are primarily junctional proteins maintaining the endothelial layer integrity [95,96]. Accompanying the loss of these proteins is a gain in mesenchymal markers like vimentin, N cadherin and α smooth muscle actin (αSMA) resulting in a polarity switch that leads to a loss of apico-basal polarity. While EndMT is characterised in tumour progression and several fibrotic diseases, wound healing also requires the coordinated transition of endothelial cells into mesenchymal populations for clot invasion and sprouting, demonstrating the complexity of this transitionary process mediating effects in homeostasis biology as well as disease pathology and progression [97]. EndMT shares many of the same marker switches that characterise the more well studied process of epithelial to mesenchymal transition or EMT. In cancer, while EMT and metastasis is reversed at the secondary sites through a process of MET or mesenchymal to epithelial transition, a reverse transition is also evident in endothelial cells. This is best described in the case of cardiac fibroblasts. Using genetic fate mapping techniques, Ubil et al. demonstrate that cardiac fibroblasts adopt endothelial-like phenotype after acute ischaemic injury [98]. While p53 pathway coordinates the Mesenchymal to endothelial (MEndoT) pathway, it remains unclear whether this is indeed a true reversal of the subset of cells that original gave rise to cardiac fibroblasts by EndMT. Indeed, a vast majority of cardiac fibroblasts originate from epithelial cells in the epicardium through an EMT process, while primarily valvular fibroblasts originate through EndMT.

## 5. Signalling in Endothelial to Mesenchymal Transition (EndMT)

**Figure 2:**
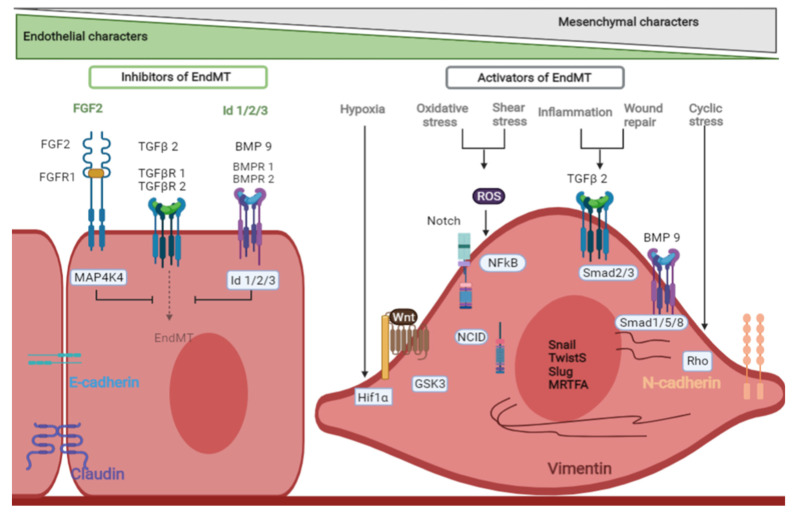
Signalling pathways that initiate and drive endothelial to mesenchymal transition (EndMT). Image produced in BioRender.

Several chemokines at the wound site are known to trigger EndMT, in particular endothelial phenotype switching triggered by hypoxia, ROS, and the pro-inflammatory milieu at the wound site, that funnels through TGFβ and BMP signalling pathways, among others, to initiate EndMT (Figure 2) [95]. Much like EMT, the transcription factors SNAIL, SLUG, Twist, MRTF-A, and ZEB proteins trigger EndMT. Notch signalling is a critical regulator of EndMT. Notch signalling promotes EndMT during development and the mutation in Notch ligand, Jagged1 (Alagille syndrome) is associated with defective EndMT dependent endocardial cushion formation in mouse [12]. The role of Notch signalling in driving EndMT was identified in early studies defining the molecular mechanisms of EndMT, albeit in in vitro cell culture conditions [99]. Indeed, foetal cell plasticity and an immature immune system enable complete regeneration of wounded tissues, which differs significantly from the wound healing in adults [100]. Therefore, developmental EndMT does not automatically indicate that the pathway promotes EndMT in adult wound healing. Additionally, the development and success of transgenic lineage tracing mouse models have allowed for further refinement of the molecular drivers of EndMT in in vivo biology. For example, recent data suggest that the functions of the Notch pathway appear to be wound stage and tissue type specific. In cutaneous wound healing, for example, a lack of Notch signalling accelerates EndMT and causes excessive scarring with delayed healing, as demonstrated by in vivo endothelial cell fate tracking in mice [13]. The endothelial cell-specific knockout of the transcriptional effector of Notch signalling, *Rbpj*, was utilised to demonstrate the EndMT acceleration in the absence of canonical Notch signalling [13]. In an additional study, Zhao et al. demonstrate that SOX9-driven EVP differentiation increases EndMT and fibrotic scarring in skin wound healing, as the SOX9 pathway acts in opposition to RBPJ–Notch signalling axis generating a double-negative feedback regulating EndMT [81]. Indeed, applying topical *Sox9* siRNA treatment directly onto wounds reduced scarring by minimising EndMT and maintaining an endothelial phenotype [81].

Additional pathways have also been identified as being important in driving EndMT in wound healing and are dependent on the specific multicellular niche with varied biochemical and microenvironmental signals at the wound site. Indeed in vitro culture causes a reduction in endothelial cell heterogeneity over time (phenotypic drift) and, therefore, the results must be substantiated in in vivo models. Notch signalling and EndMT transitioning properties are clearly subject to phenotypic drift in culture. Interestingly, there is more robustness in TGFβ signalling-driven EndMT suggesting that this pathway has more built-in redundancy to enable state transitions overcoming in vitro monoculture-driven drift. In vitro and in vivo modelling using numerous pathological scenarios of EndMT, are promoted via biochemical and environmental cues such as Semaphorin 7A, hypoxia, inflammatory cytokines, ROS and cyclical strain function, that drive EndMT through the TGFβ–Smad signalling axis [101,102,103,104,105,106,107]. Downstream of TGFβ signalling are various independent mechanisms that enforce the mesenchymal state like Snail expression, MKL1 dependent Twist1 expression, and actin reorganisation [108,109,110]. This network of effectors and positive feedback loops ensure that the cells progress towards EndMT upon TGFβ activation. Twist1, an EndMT transcription factor downstream of TGFβ, is also known to increase levels of TGFβ receptor 2 (TGFβRII) and thereby phospho-Smad2 levels, establishing a positive feedback for EndMT [111,112]. As a critical node determining EndMT, the TGFβ pathway is subjected to several checks and balances to prevent unwarranted EndMT. Indeed, murine cell lineage tracing also reveals the role of the TGFβ–Smad2 axis in EndMT in vivo. Cooley et al. elegantly demonstrated that endothelial specific *Smad2* knockout and *Smad3* haploinsufficiency resulted in reduced EndMT in venous grafts preventing postoperative stenosis and scarring, crucial in defining better treatments for patients undergoing vascular graft surgery [111].

FGF signalling and inhibitor of DNA proteins (Id proteins) are among the most notable pathways countering TGFβ-driven EndMT [113,114,115]. FGF2 increases miR20 expression which targets ALK5 and TGFβRII that transduce TGFβ signalling thereby inhibiting EndMT [116]. A recent study by Ma et al. demonstrated the role of Id proteins (Id1, Id2 and Id3) in repressing the function of both SNAIL and SLUG, blocking TGFβ-driven EndMT, as well as being essential in ensuring endothelial phenotype is maintained [115]. Wnt signalling is a key regulator of EndMT. In response to myocardial injury, lineage-tracing experiments demonstrate that canonical Wnt high and SMA+ mesenchymal cells are derived from endothelial cells contributing to cardiac tissue repair [117]. Wnt-3a driven EndMT of human dermal microvascular endothelial cell contributes to dermal fibrosis [118]. Overall, these data demonstrate the complexity of the molecular drivers of EndMT in a wound environment and therefore heighten the importance for future in vivo studies to be conducted to elucidate novel mechanisms, whereby therapies can be developed to prevent excessive scarring developing due to aberrant EndMT.

## 6. Cytoskeletal Reorganisation in Endothelial to Mesenchymal Transition (EndMT)

Regulation of actin dynamics is essential in EndMT. All the multiple steps of wound healing such as haemostasis, clot formation and fibrin invasion by endothelial cells, migration on provisional matrix and finally lumenisation and stabilisation of endothelial sprouts to close the wound require coordination of the actin cytoskeletal dynamics. The RhoGTPase family of proteins, particularly RhoA and CDC42, coordinate EndMT downstream of the TGFβ signalling pathway [110]. Another RhoGTPase, RhoJ, an endothelium enriched GTPase, is implicated in hypoxia-induced EndMT [119]. RhoJ promotes transcriptional repression of the junction VE cadherins by the transcription factors SNAIL and Twist1. RhoJ is also implicated in regulating the remodelling of the provisional fibronectin matrix [120]. Thus, coordinated regulation of RhoGTPases by upstream signalling cascades regulates EndMT.

Mechanisms that couple these actin cytoskeletal rearrangements to main signalling pathways are also crucial in the process of EndMT. NCK1/2 is one such key adapter protein, linking surface receptors like VEGFRs to actin cytoskeletal modulators like RAC1 and PAK kinases. *Nck* endothelial knockout mice die during early embryonic development (E10) due to defective EndMT and cardiac valve morphogenesis, showing that *Nck* plays a critical role in developmental EndMT. Furthermore, *Nck* null endothelia are defective in migration downstream of VEGF and angiopoietin signalling, suggesting that *Nck*-driven cytoskeletal reorganisation aids in endothelial migration during wound healing [121]. By regulating the establishment of endothelial front-rear polarity, which is essential for sprouting angiogenesis to occur correctly, NCK promotes endothelial migration downstream of VEGF signalling. Indeed, *Nck* knockout mice also show reduced pathological angiogenesis in oxygen-induced retinopathy models and delayed cutaneous wound healing due to defective sprouting angiogenesis [122]. Therefore, better understanding of the drivers of endothelial cytoskeleton reorganisation and the molecular mechanism behind this process is another significant pathway in better understanding pathological EndMT.

## 7. Receptor Trafficking in Endothelial Heterogeneity

VEGF is the key mitogen and chemotactic factor secreted by fibroblasts, smooth muscle cells, platelets, neutrophils, macrophages and endothelial cells in the wound [123]. Response to VEGF is coordinated by VEGF receptors 1–3. Sprouting endothelial cells at the vascular front have high levels of VEGF uptake, receptor endocytosis and turnover while stable, quiescent vessel endothelium has reduced receptor endocytosis. The trafficking of VEGFR2 and VEGFR3 is regulated by the clathrin-associated adapter protein DAB2, which is inhibited by aPKC-mediated phosphorylation in stable vessels. Presumably, the quiescent endothelial cells have a higher junctional stability that allows for cadherin-dependent recruitment and sustained phosphorylation and activation of aPKC that allows these endothelial cells to reduce VEGFR2/3 turnover, thereby maintaining the quiescent cell fate [124]. This suggests that, in addition to a VEGF gradient that drives sprouting, trafficking heterogeneity also dictates the acquisition of distinct cell fates and behaviours by endothelial cells.

In addition to a regulation of the surface VEGFR2 receptor, the Golgi pool of VEGFR2 that is rapidly recruited to the plasma membrane in response to VEGF is also a critical player in angiogenesis. Syntaxin 6 maintains this pool and, in the absence of this protein, the VEGF receptors are targeted to lysosomes for degradation. Cells show impaired wound healing migration in response to VEGF in the absence of the reserve pool of receptors in the Golgi maintained by syntaxin6 [125]. Thus, receptor trafficking, both onwards in the secretory pathway as well as receptor internalisation and degradation play a key role in determining endothelial cell behaviour.

Notch receptor trafficking also plays an important role in controlling endothelial cell heterogeneity, with Notch ligand DLL4 stimulating Notch receptor cleavage by γ-secretases. This process is also regulated by receptor endocytosis, where the incorporation of γ-secretase and Notch receptors together enables the cleavage and signalling to take place from endosomes. The lack of the class II α-isoform of phosphatidylinositol 3-kinase (PI3K C2α) causes a failure to internalise γ-secretase with the accumulation of unprocessed receptors in intracellular vesicles, thereby inhibiting the Dll4-driven EndMT in vitro [126]. While regulators of receptor endocytosis and pairing of receptors to endosomal substrates remarkably alter downstream signalling and endothelial cell properties, further evidence for the endosomal signalling of VEGFR2 and Notch pathways in vivo in endothelial cell state transitions remains a challenge in the field.

## 8. Metabolic Plasticity and Heterogeneity of Endothelial Cells

Wound healing angiogenesis is an energetically demanding process that causes endothelial metabolic reprogramming. The endothelial cells respond by dramatically increasing both glycolytic flux as well as mitochondrial respiration. Acquiring distinct metabolic phenotypes in quiescent versus angiogenic vasculature is necessary to meet the bioenergetic demands of rapid proliferation and motility during angiogenesis. The COX family of proteins are crucial in the metabolic pathway of endothelial cells. For example, deletion of the *Cox10* gene specifically in endothelial cells leads to respiratory insufficiency and embryonic lethality. Interestingly, deletion of this gene in adults delays wound healing responses [127]. Along similar lines, pathogenetic plasticity of endothelial cells and acquisition of a hyper glycolytic state during healing of burns could lead to abnormal scars such as keloids [128]. Targeting aberrant glucose metabolism in endothelial cells through a PKM2 inhibitor improves wound healing in vivo [128]. Thus, the metabolic phenotypes and their heterogeneity in endothelial cells is a key regulator of wound healing. Indeed, single cell metabolic imaging reveals metabolic heterogeneity at sub-cellular resolution. Motile endothelial cells depend on a burst of glycolysis that is coupled to actin reorganisation. RhoA-ROCK driven trafficking of GLUT3 serves to fuel endothelial cell contractility [129]. Several studies now indicate that endothelial cell motility depends on glycolysis while proliferation depends on mitochondrial oxidative phosphorylation [130,131]. Targeting endothelial cell metabolic switching and limiting endothelial cell plasticity is currently explored as a novel approach to reduce excessive angiogenesis in tumours and the same can be beneficial in wound repair, with future studies required to explore the modulation of metabolic plasticity as a therapeutic strategy.

## 9. Conclusions

In this review, we have summarised the key phenotypes and mechanisms that govern endothelial heterogeneity and plasticity with specific focus on wound healing. Importantly, we have highlighted the roles of endothelial diversity in leading vascular network formation during foetal development and in regeneration during wound healing. However, despite these important findings there is still significant work to be undertaken to continue to elucidate the molecular drivers of endothelial function, particularly in disease progression in the adult system. Future studies will be needed to continue to unravel the complexity of endothelial biology, which will provide excellent pathways for novel therapeutic development in treating vascular disease and scarring in wound healing.

## Data Availability

No new data were created or analysed in this study.
